# Disseminated Nocardia otitidiscaviarum Presenting as Lung-Brain-Skin Syndrome in a Renal Transplant Recipient: A Case Report

**DOI:** 10.7759/cureus.115600

**Published:** 2026-09-01

**Authors:** Enas Abdallah, Mostafa M Abouzahra, Aakash Rai, Mehakmeet Bhatia, Vrajesh Parmar, Shamik Parikh, Chidamber B Alamelumangapuram, Vivek Kak

**Affiliations:** 1 Malignant Hematology, Moffitt Cancer Center, Tampa, USA; 2 Faculty of Medicine, Al-Azhar University, Cairo, EGY; 3 General Practice, Liaquat University of Medical and Health Sciences, Jamshoro, PAK; 4 Infectious Diseases, Detroit Medical Center, Detroit, USA; 5 Internal Medicine, Northwest Primary Care at Houghton, Tucson, USA; 6 Internal Medicine, Baptist Medical Center, San Antonio, USA; 7 Internal Medicine, Henry Ford Jackson Hospital, Jackson, USA; 8 Infectious Disease, Henry Ford Jackson Hospital, Jackson, USA

**Keywords:** brain abscess, disseminated nocardiosis, immunosuppression, lung-brain-skin syndrome, nocardia otitidiscaviarum, pulmonary nocardiosis, renal transplant, soft tissue abscess

## Abstract

We present a case of disseminated *Nocardia otitidiscaviarum* infection in a 72-year-old male with a history of renal transplantation on chronic immunosuppressive therapy with prednisone, cyclosporine, and mycophenolate mofetil. He presented with pneumonia and was initially treated with ceftriaxone and azithromycin. Bronchoalveolar lavage (BAL) and cultures from a biopsied thigh lesion both grew *N. otitidiscaviarum*. His hospital course was complicated by visual hallucinations, and brain magnetic resonance imaging (MRI) revealed multiple intracranial abscesses. He initially received meropenem, trimethoprim-sulfamethoxazole (TMP-SMX), and linezolid. His antimicrobial regimen was subsequently modified during the treatment course. Linezolid was discontinued after hematologic abnormalities developed, TMP-SMX was temporarily held because of worsening renal function and later reintroduced, and meropenem was also discontinued. He was discharged on TMP-SMX and minocycline and completed an initial six-week antimicrobial treatment course from the time of diagnosis. He was subsequently maintained on minocycline and moxifloxacin; moxifloxacin was later discontinued because of QT-interval prolongation, while minocycline was continued for slightly longer than one year, with clinical and radiologic resolution.

## Introduction

*Nocardia* is a genus of aerobic, Gram-positive bacteria that are ubiquitously found in the environment. Primary exposure occurs through inhalation of contaminated soil particles. Most *Nocardia* infections occur in patients with impaired T-cell-mediated immunity. *Nocardia* primarily affects patients with solid organ or hematopoietic stem cell transplantation, acquired immunodeficiency syndrome (AIDS), or chronic glucocorticoid use [1-4].

Disseminated disease presenting as lung-brain-skin syndrome is uncommon [1,3]. Pulmonary infection is the most common presentation of nocardiosis, while hematogenous dissemination may involve the central nervous system (CNS) and skin; CNS involvement is particularly important because it is associated with substantial morbidity and may occur even in the absence of prominent neurologic symptoms [1,2].

Infections caused by *Nocardia otitidiscaviarum* are rare and may exhibit variable antimicrobial susceptibility, which can complicate therapeutic selection. Primary cutaneous nocardiosis may also be caused by other, newly characterized *Nocardia* species [5]. Here, we describe a case of disseminated nocardiosis caused by *N. otitidiscaviarum* in a renal transplant recipient and present a focused literature review of previously reported adult disseminated infections caused by this species, highlighting the diagnostic and therapeutic challenges posed by disseminated *N. otitidiscaviarum* infection in a solid-organ transplant recipient, particularly the importance of recognizing multisystem involvement and tailoring therapy to susceptibility results.

## Case presentation

A 72-year-old man presented with several days of pleuritic chest pain, shortness of breath, productive cough, fever, and chills. His medical history was significant for renal transplantation secondary to granulomatosis with polyangiitis approximately 21 years earlier. He was receiving chronic immunosuppressive therapy with prednisone, cyclosporine, and mycophenolate mofetil. He denied recent travel, sick contacts, changes to his immunosuppressive regimen, prior graft rejection, HIV infection, and chronic airway disease. He reported past travel to Vietnam around the 1970s, with possible soil exposure; however, the exact interval between this travel and the current illness could not be determined from the available documentation. He was a former smoker with a 10-pack-year smoking history.

On presentation, he was tachycardic, febrile to 101.2°F, tachypneic, and normotensive. He initially required 2 L/minute of supplemental oxygen via nasal cannula, which was new for him, with subsequent escalation to a maximum of 4 L/minute. He did not require noninvasive ventilation or endotracheal intubation. Laboratory evaluation revealed leukocytosis with a white blood cell count of 19,000/µL and neutrophilic predominance. Initial chest radiography (Figure 1) and computed tomography (CT) (Figure 2) demonstrated pulmonary infiltrates predominantly involving the left lower lobe.

**Figure 1:**
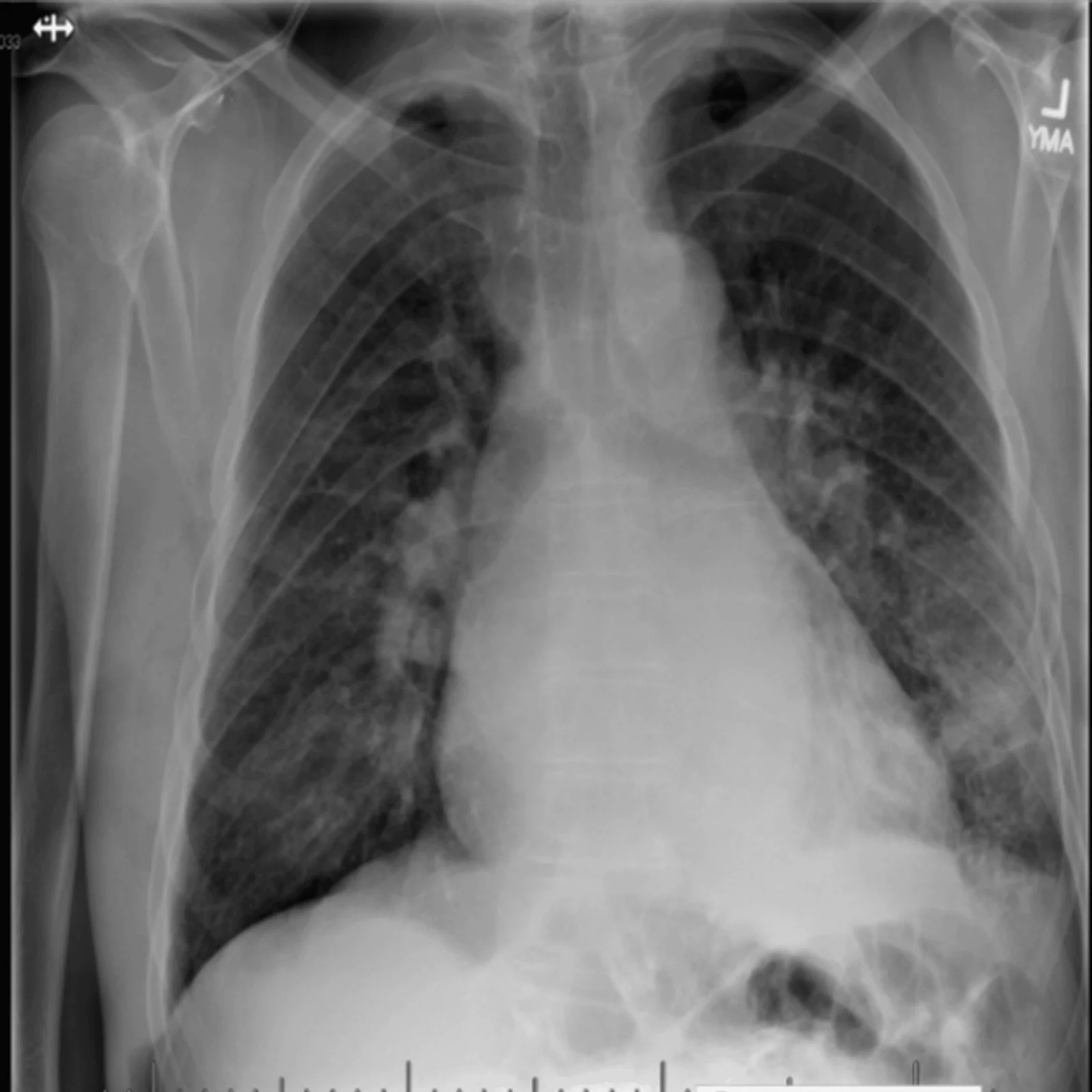
Initial chest radiograph demonstrating left basilar air-space consolidation

**Figure 2:**
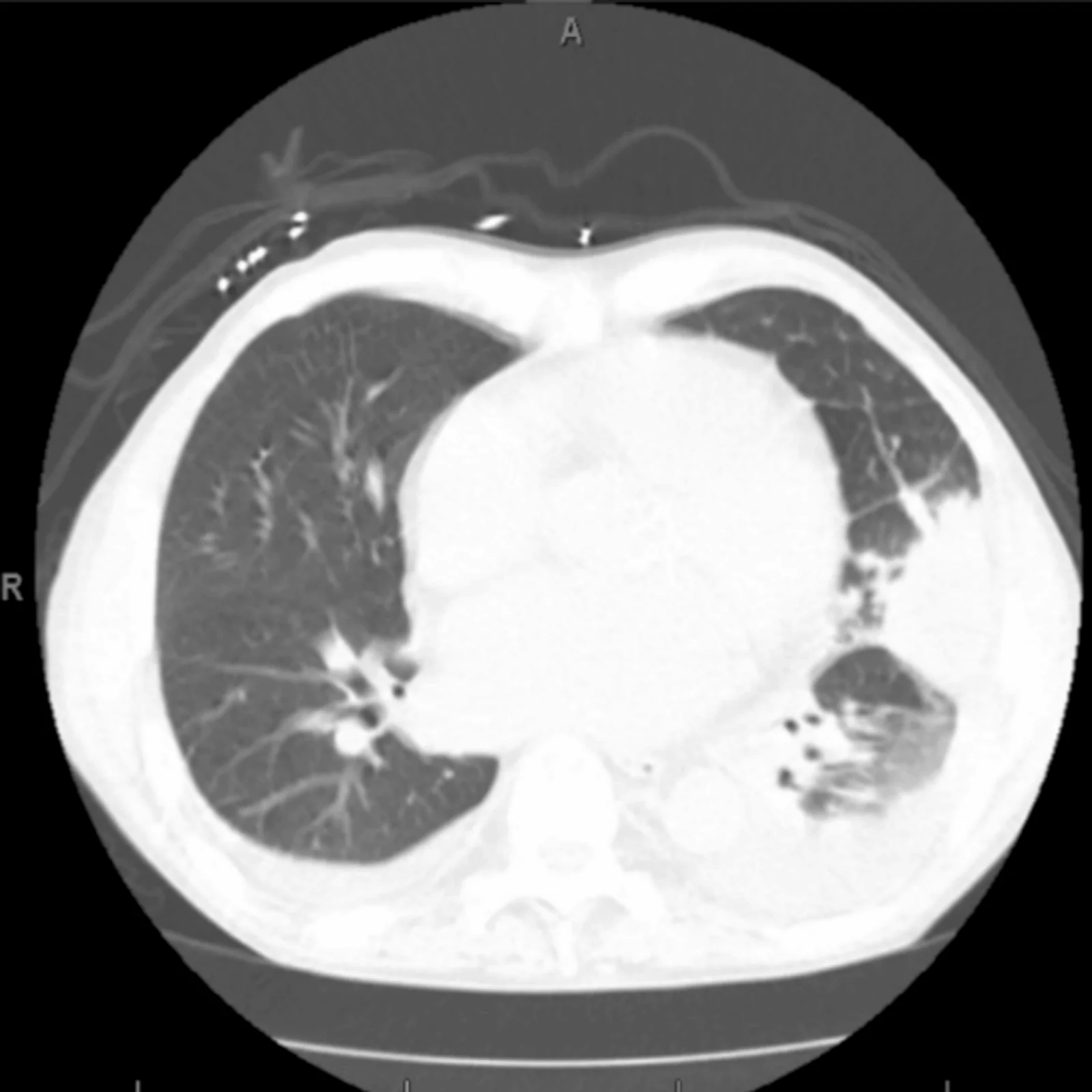
Initial chest CT (axial view) without contrast demonstrating left lower lobe consolidation with a small left pleural effusion

Empiric treatment for community-acquired pneumonia was initiated with ceftriaxone 2 g daily and azithromycin 500 mg daily. Cyclosporine and prednisone were continued to reduce the risk of graft rejection. Because of worsening hypoxemia and progressive pulmonary opacities, antimicrobial coverage was broadened to linezolid and cefepime. Urinary antigens for *Legionella pneumophila* and *Streptococcus pneumoniae* were negative. Repeat chest CT demonstrated worsening left-sided consolidations (Figure 3) and patchy right-sided pulmonary opacities (Figure 4).

**Figure 3:**
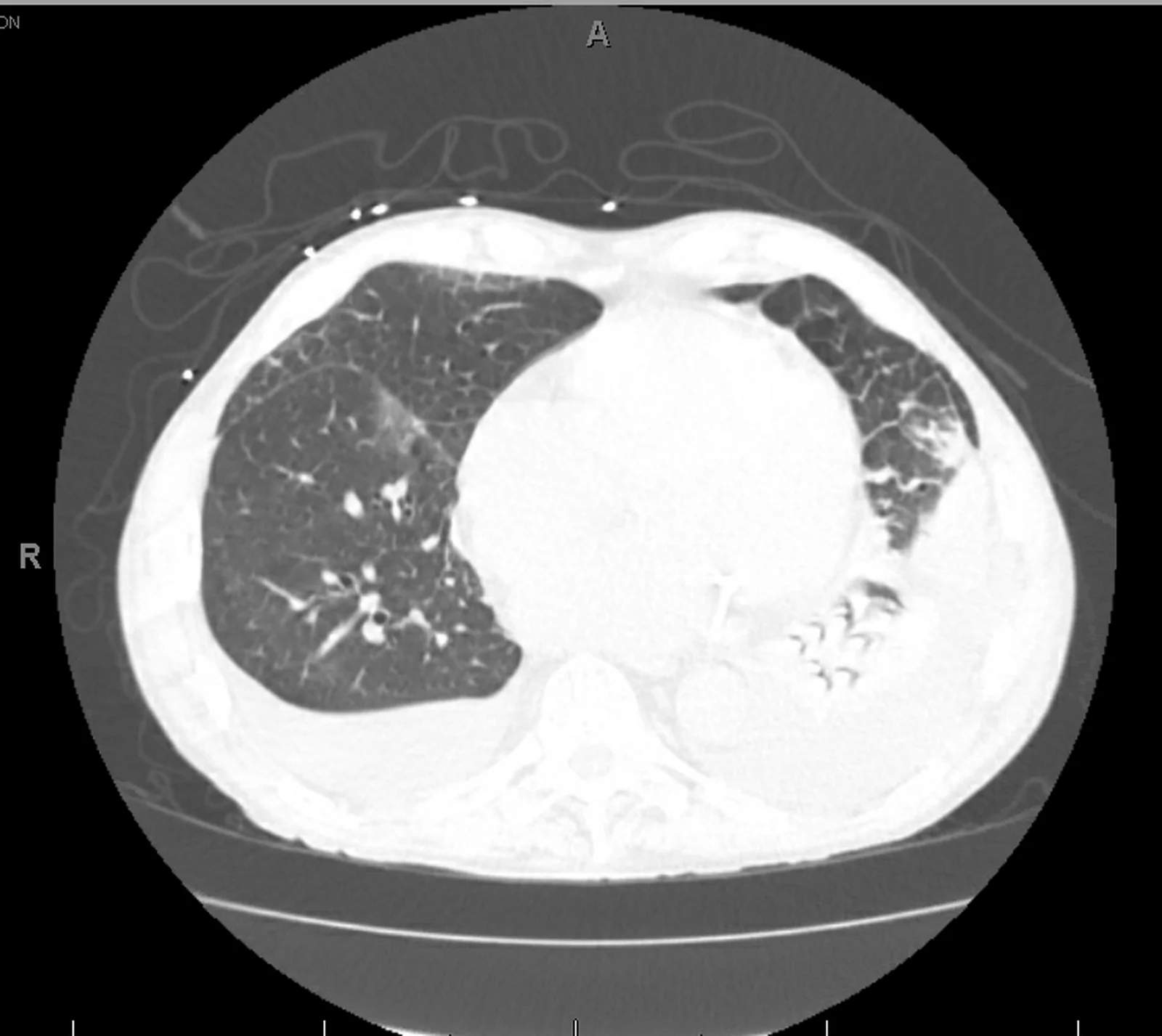
Follow-up chest CT (axial view) demonstrating worsening left-sided pulmonary consolidation

**Figure 4:**
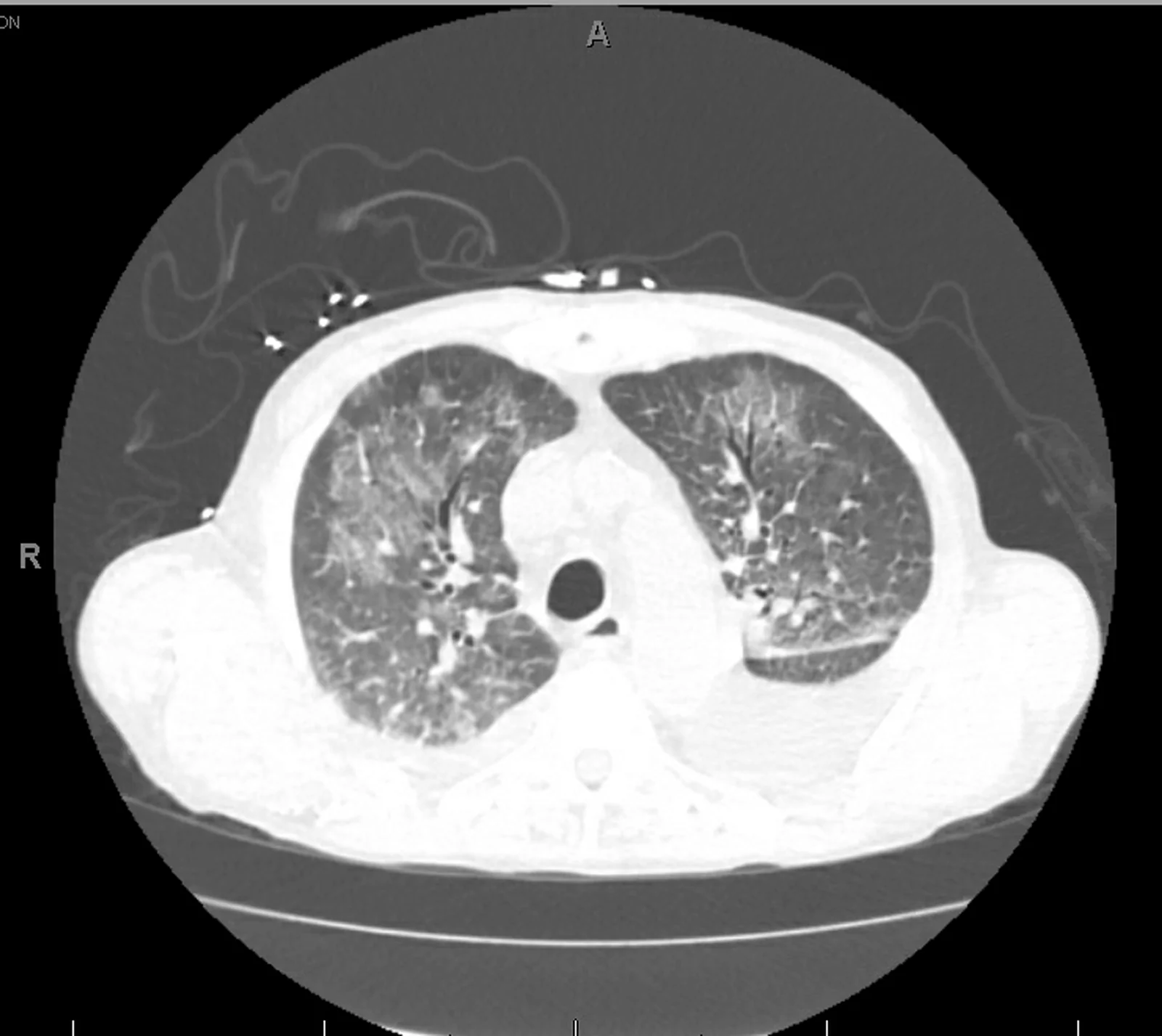
Follow-up chest CT (axial view) demonstrating patchy right-sided pulmonary opacities

Approximately one week after admission, two lower-extremity soft tissue lesions were identified, including a thigh lesion and a tender, purulent collection above the right ankle (Figure 5).

**Figure 5:**
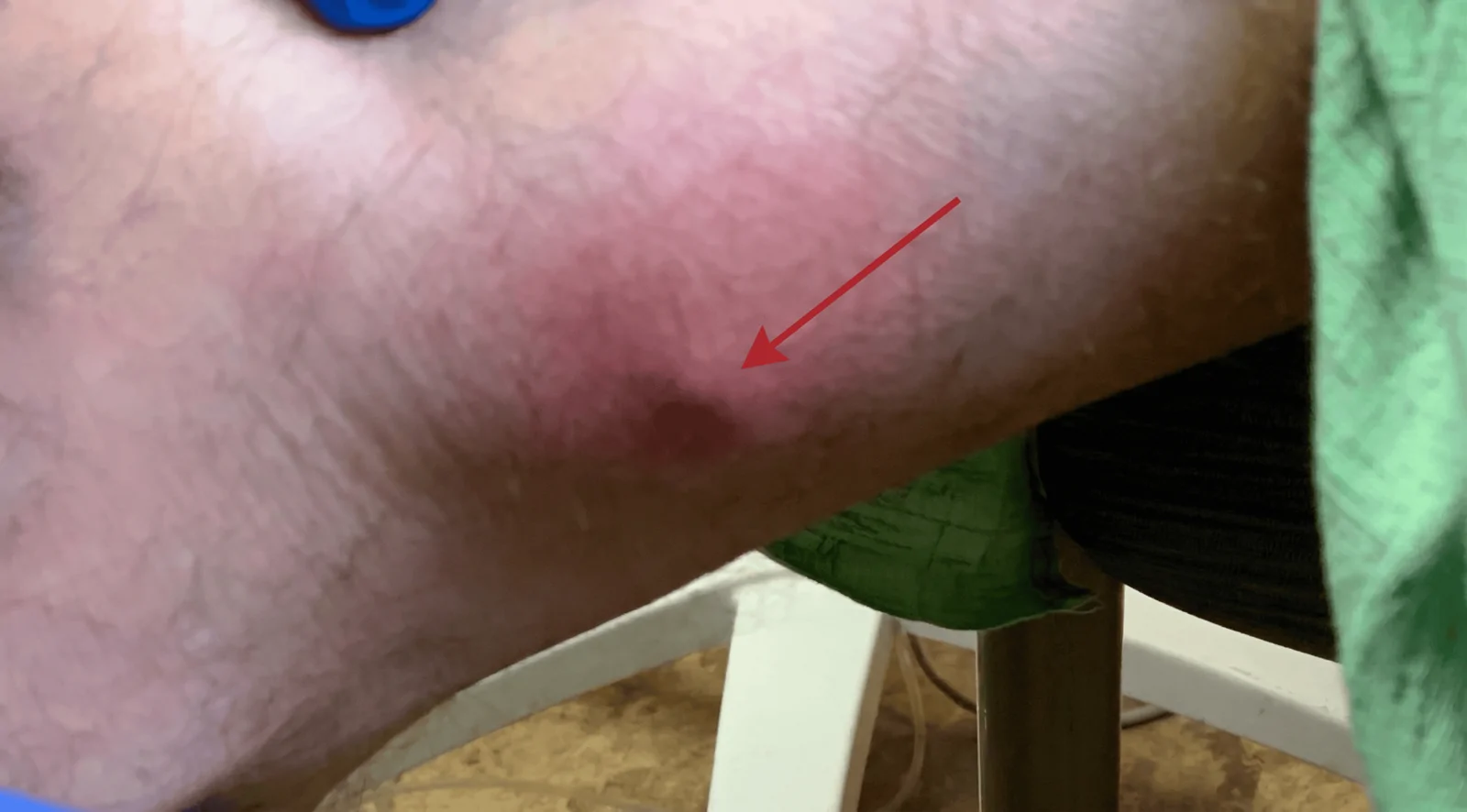
Right lower-extremity soft tissue lesion (arrow) with surrounding erythema

The patient was unable to determine how long the lesions had been present but reported recent worsening. The thigh lesion was biopsied, and tissue cultures grew *N. otitidiscaviarum* after 48 hours. Empiric antimicrobial therapy was changed to linezolid and imipenem. Because of persistent hypoxemia, bronchoscopy with bronchoalveolar lavage (BAL) was performed, and BAL cultures also grew *N. otitidiscaviarum*. Modified acid-fast staining of the BAL and wound specimens demonstrated filamentous branching bacteria. For species-level identification, the BAL isolate was sent to the Centers for Disease Control and Prevention (CDC) laboratory, where *N. otitidiscaviarum* was identified using matrix-assisted laser desorption/ionization time-of-flight (MALDI-TOF) mass spectrometry. Testing for cytomegalovirus, *Blastomyces*, *Coccidioides*, *Pneumocystis jirovecii*, and respiratory viruses was negative.

On hospital day 12, the patient developed confusion with visual and auditory hallucinations. Magnetic resonance imaging (MRI) of the brain with and without contrast demonstrated multiple enhancing lesions involving the right cerebellar peduncle, right occipital lobe, right temporal lobe, and bilateral parietal lobes, concerning for intracranial abscesses (Figure 6).

**Figure 6:**
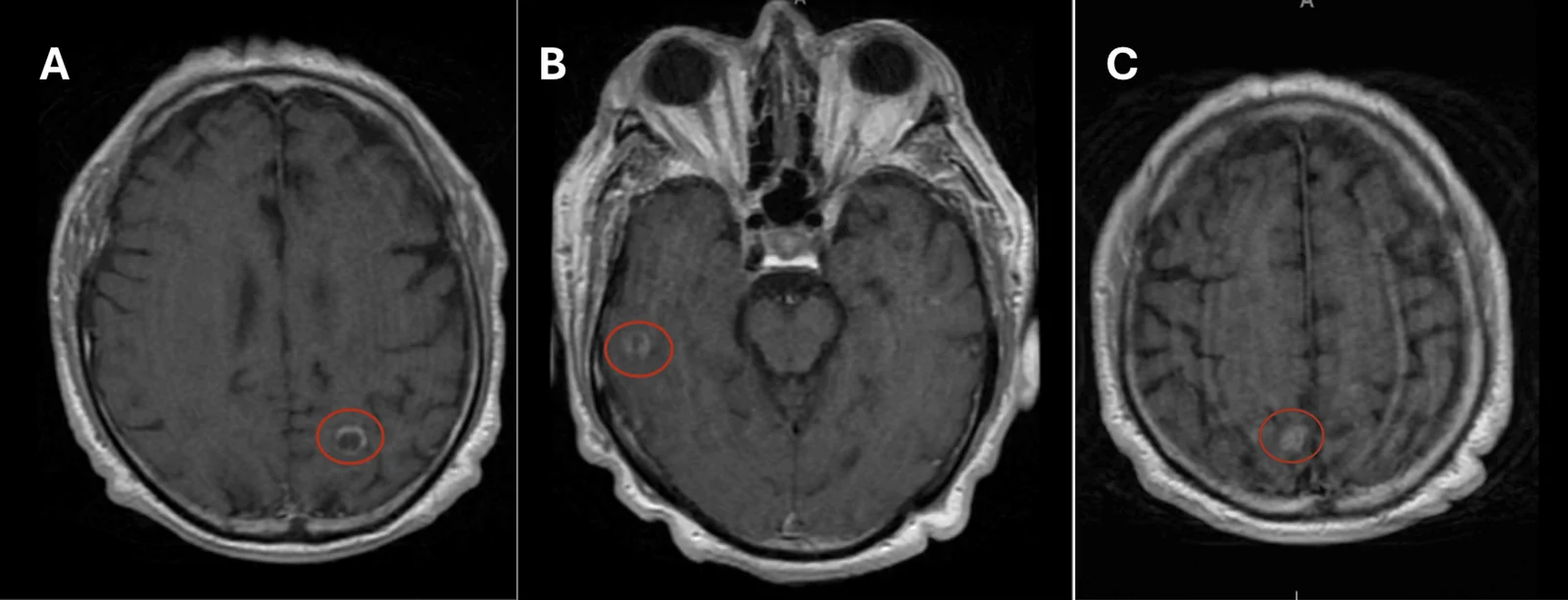
Contrast-enhanced brain MRI (axial view) demonstrating multiple enhancing intracranial lesions (red circles) concerning for abscesses

Lumbar puncture was not performed, and CNS involvement was diagnosed based on the MRI findings. CT of the abdomen and pelvis showed no additional abscesses. Because of disseminated disease with CNS involvement, imipenem was changed to meropenem for improved CNS penetration, and trimethoprim-sulfamethoxazole (TMP-SMX) was added to linezolid.

BAL and abscess isolates were sent to the CDC laboratory for susceptibility testing. The organism was susceptible to TMP-SMX, amikacin, linezolid, and minocycline and resistant to amoxicillin-clavulanate, ceftriaxone, imipenem, and ciprofloxacin (Table 1).

**Table 1:** Antimicrobial susceptibility profile of Nocardia otitidiscaviarum Antimicrobial susceptibility testing of the *N. otitidiscaviarum* isolate was performed at the Centers for Disease Control and Prevention laboratory. Numerical minimum inhibitory concentration (MIC) values, the specific susceptibility-testing methodology, and meropenem susceptibility were not available in the source documentation.

Antimicrobial	Susceptibility Interpretation
Amikacin	Susceptible
Tobramycin	Susceptible
Ceftriaxone	Resistant
Clarithromycin	Resistant
Minocycline	Susceptible
Trimethoprim-sulfamethoxazole	Susceptible
Imipenem	Resistant
Amoxicillin-clavulanate	Resistant
Moxifloxacin	Intermediate
Ciprofloxacin	Resistant
Linezolid	Susceptible
Vancomycin	Resistant

Key laboratory and microbiological findings during hospitalization and readmission are summarized in Table 2. 

**Table 2:** Key laboratory and microbiological findings during hospitalization and readmission BAL: bronchoalveolar lavage; MALDI-TOF: matrix-assisted laser desorption/ionization time-of-flight.

Investigation	Result
White blood cell count at presentation	19,000/µL with 91% neutrophils
*Legionella pneumophila* urinary antigen	Negative
*Streptococcus pneumoniae* urinary antigen	Negative
Thigh lesion tissue culture	Grew *Nocardia otitidiscaviarum*
Bronchoalveolar lavage culture	Grew *N. otitidiscaviarum*
Modified acid-fast staining of BAL and wound specimens	Demonstrated filamentous branching bacteria
Species-level identification of BAL isolate	*N. otitidiscaviarum* identified by MALDI-TOF mass spectrometry at the Centers for Disease Control and Prevention laboratory
Cytomegalovirus testing	Negative
*Blastomyces* testing	Negative
*Coccidioides* testing	Negative
*Pneumocystis jirovecii* testing	Negative
Respiratory viral testing	Negative
BAL galactomannan	Positive
Serum galactomannan	Negative
Fungitell assay	Negative
Hemoglobin on readmission	6.3 g/dL, decreased from 7.7 g/dL
Platelet count on readmission	71,000/µL, decreased from 103,000/µL

During therapy, TMP-SMX was discontinued after approximately one week because of worsening renal function. The patient was discharged on linezolid and meropenem but returned to the hospital one week later with worsening anemia and thrombocytopenia. Hemoglobin had decreased from 7.7 to 6.3 g/dL, and the platelet count had decreased from 103,000/µL to 71,000/µL. Evaluation revealed no evidence of active gastrointestinal bleeding, disseminated intravascular coagulation, or thrombotic thrombocytopenic purpura. Linezolid was considered the likely cause of the hematologic abnormalities and was discontinued.

After renal function improved, TMP-SMX was reintroduced 15 days after the last dose. Meropenem was subsequently discontinued during the treatment course. The patient was discharged on TMP-SMX and minocycline and completed an initial six-week antimicrobial treatment course from the time of diagnosis. He was subsequently maintained on minocycline and moxifloxacin; moxifloxacin was discontinued after approximately five months because of QT-interval prolongation, with a corrected QT interval of 563 ms while he was receiving other QT-prolonging medications, including amiodarone. Minocycline was continued for slightly longer than one year. Follow-up chest CT and brain MRI approximately one year later demonstrated resolution of the disseminated lesions, with no clinical recurrence.

The patient was also treated for suspected pulmonary aspergillosis with anidulafungin followed by isavuconazole for six weeks after BAL galactomannan testing was positive, while serum galactomannan and Fungitell assays were negative. No tissue culture or polymerase chain reaction (PCR) testing for *Aspergillus* was obtained. Table 3 provides a chronological summary of the patient’s clinical course.

**Table 3:** Chronological summary of the patient’s clinical course BAL: bronchoalveolar lavage; CNS: central nervous system; CT: computed tomography; MRI: magnetic resonance imaging; TMP-SMX: trimethoprim-sulfamethoxazole.

Time Point	Clinical findings/Investigations	Management/Outcome
Presentation	Pleuritic chest pain, shortness of breath, productive cough, fever, and chills. Chest radiography and CT demonstrated predominantly left lower-lobe pulmonary infiltrates.	Empiric ceftriaxone and azithromycin were initiated.
Early hospitalization	Worsening hypoxemia and progressive pulmonary opacities.	Antimicrobial coverage was broadened to linezolid and cefepime.
Approximately one week after admission	Two lower-extremity soft-tissue lesions were identified. The thigh lesion was biopsied, and tissue culture grew *N. otitidiscaviarum.*	Antimicrobial therapy was changed to linezolid and imipenem.
During hospitalization	Bronchoscopy with BAL was performed because of persistent hypoxemia. BAL culture also grew *N. otitidiscaviarum*.	Pulmonary and cutaneous microbiologic evidence supported disseminated nocardiosis.
Hospital day 12	Confusion with visual and auditory hallucinations developed. Brain MRI demonstrated multiple enhancing intracranial lesions concerning for abscesses.	Imipenem was changed to meropenem for improved CNS penetration, and TMP-SMX was added to linezolid.
During therapy	TMP-SMX was discontinued after approximately one week because of worsening renal function. One week after discharge, hemoglobin decreased from 7.7 to 6.3 g/dL and platelet count from 103,000/µL to 71,000/µL.	Linezolid was considered the likely cause of the hematologic abnormalities and was discontinued. TMP-SMX was reintroduced 15 days after the last dose once renal function improved.
Initial treatment period	—	Meropenem was discontinued, and the patient was discharged on TMP-SMX and minocycline, completing a total of six weeks of antimicrobial therapy from the initial diagnosis.
Maintenance phase	—	Minocycline and moxifloxacin were used for maintenance therapy. Moxifloxacin was discontinued after approximately five months because of QT-interval prolongation.
Approximately one year later	Follow-up imaging demonstrated resolution of the disseminated lesions, with no clinical recurrence.	Minocycline had been continued for slightly longer than one year.

## Discussion

Literature review

*N. otitidiscaviarum*, previously known as *Nocardia caviae*, is an uncommon species of *Nocardia* associated with pulmonary, cutaneous, central nervous system, and disseminated infection. In a systematic review published by in 2026, Alekrish et al. [6] searched PubMed/MEDLINE, Web of Science, and ScienceDirect from database inception through December 2023 using the strategy ("Nocardia otitidiscaviarum" OR otitidiscaviarum) AND ("disseminated nocardiosis" OR "systemic nocardiosis" OR "extrapulmonary nocardiosis") and identified nine adult cases of disseminated *N. otitidiscaviarum* infection [7-15]. Importantly, that strategy did not include the historical synonym *Nocardia caviae* and required terms related to disseminated, systemic, or extrapulmonary nocardiosis; therefore, the nine cases cannot be said to be a complete enumeration of all historically reported cases.

To identify reports after December 2023, we performed an updated PubMed search on August 26, 2026, using the search strategy "Nocardia otitidiscaviarum" OR "Nocardia caviae". Because this search was intended as an update to the published systematic review by Alekrish et al. [6] rather than a de novo reassessment of the pre-2024 literature, only reports published from January 1, 2024, through August 26, 2026, were assessed for inclusion in the updated case summary if they met our definition of adult disseminated infection. Four additional reports meeting this focused definition were identified during the update period [16-19]. Disseminated disease was considered present when at least two noncontiguous anatomical sites were involved in a pattern consistent with hematogenous spread or when the authors explicitly described disseminated disease supported by multisite involvement.

Across the published cases summarized in Table 4, pulmonary and CNS involvement were prominent, while cutaneous, renal, hepatic, thyroid, and other extrapulmonary sites were also reported. Relevant host factors included chronic corticosteroid exposure, systemic autoimmune disease, malignancy receiving chemotherapy, renal disease, and other chronic comorbidities, although disseminated infection was also reported in patients described as immunocompetent. Antimicrobial regimens were heterogeneous and frequently required multidrug therapy and modification according to susceptibility results, clinical response, or treatment-related toxicity. Outcomes ranged from clinical and radiologic resolution to death, underscoring the potential severity of disseminated *N. otitidiscaviarum* infection.

**Table 4:** Summary of published adult disseminated Nocardia otitidiscaviarum cases up to August 26, 2026 “Not reported” indicates that the corresponding information could not be established from the sources used for this table. TMP-SMX: trimethoprim-sulfamethoxazole; IV: intravenous; CNS: central nervous system; COPD: chronic obstructive pulmonary disease; ESRD: end-stage renal disease; CKD: chronic kidney disease; M: male; F: female

Study (Author, Year)	Age/Sex	Immunocompromising condition/Relevant host factor	Clinical sites involved	Antimicrobial treatment	Susceptibility profile, when available	Treatment Duration	Outcome
Hemmersbach-Miller et al., 2004 [7]	Not reported	COPD, renal transplant, diabetes mellitus	Lung, skin, brain	TMP-SMX, ofloxacin, ciprofloxacin, ceftazidime, vancomycin; surgical drainage of intracranial abscesses	Not reported	147 days	Died
Sharma et al., 2007 [8]	36 / F	Sickle cell anemia, ESRD, dilated cardiomyopathy, hypertension	Lung, blood	IV TMP-SMX followed by IV amikacin, oral gatifloxacin, and oral TMP-SMX	Not reported	Not reported	Infection eradicated
Jiang et al., 2016 [9]	37 / M	Described as immunocompetent; subclinical hypothyroidism	Lung, skin/soft tissue, liver	Ceftriaxone + TMP-SMX, followed by TMP-SMX + minocycline	Not reported	4 months	Resolved
Zheng, 2019 [10]	59 / M	Hypertension, dyslipidemia, stage IV CKD; prednisolone exposure	Lung, soft tissue/skin, brain	Imipenem + TMP-SMX → TMP-SMX + linezolid + tigecycline → maintenance TMP-SMX + moxifloxacin	Susceptibility testing performed; complete profile not reported in the source summary used for this table	6 weeks of combination therapy followed by maintenance	Resolved
Talon et al., 2021 [11]	30 / F	Hepatic impairment	Liver, lung, brain	IV TMP-SMX + intrathecal amikacin; external ventricular drainage	Not reported	Not reported	Died
Parengal et al., 2021 [12]	29 / F	Systemic lupus erythematosus with multiorgan involvement	Lung, skin, brain; additional visceral involvement reported	IV meropenem + TMP-SMX + amikacin → oral moxifloxacin + TMP-SMX	Susceptible: TMP-SMX, amikacin, ciprofloxacin, moxifloxacin, linezolid; resistant: amoxicillin-clavulanate, ceftriaxone, clarithromycin	8 weeks IV followed by planned 10 months oral therapy	Brain abscesses resolved; poor neurologic outcome
Barry et al., 2022 [13]	37 / F	Stage IV metastatic breast cancer	Skin, lung, brain	TMP-SMX + imipenem-cilastatin initially; subsequently amikacin, linezolid, moxifloxacin, and doxycycline	Susceptible: amikacin, linezolid; resistant: TMP-SMX, ciprofloxacin, imipenem, cefepime, cefoxitin, amoxicillin-clavulanate, clarithromycin, tobramycin	22 days	Died
Kullab et al., 2022 [14]	36 / F	Stage IV metastatic breast cancer receiving chemotherapy	Lung, skin, brain	Amikacin + linezolid + ceftriaxone; moxifloxacin added after 5 weeks	Susceptible: amikacin, linezolid; resistant: TMP-SMX, ciprofloxacin, imipenem, cefepime, amoxicillin-clavulanate, tobramycin, clarithromycin	>9 weeks	Improved; infection not completely eradicated
Duangprasert et al., 2022 [15]	51 / M	Autoimmune hepatitis	Lung, brain	TMP-SMX + amikacin + moxifloxacin; bifrontal decompressive craniectomy	Susceptible: TMP-SMX, amikacin, moxifloxacin; resistant: imipenem	Not reported	Died from septic shock
Huang and Jia, 2024 [16]	Not reported	Described as immunocompetent; COPD	Lung, skin, intracranial lesions	Low-dose TMP-SMX + imipenem-cilastatin	Not reported	Not reported	Favorable outcome
Zhang et al., 2024 [17]	66 / M	Minimal change disease; long-term high-dose prednisone	Lung, skin, CNS	Linezolid + TMP-SMX	Susceptibility testing performed by Kirby-Bauer method; complete isolate-specific categorical profile not reported	Not reported	Pathogen eradication reported
Zhang et al., 2026, (Case 3) [18]	78 / F	Described as immunocompetent; hypertension and prior cerebral infarction	Lung, brain, bilateral kidneys	Piperacillin-tazobactam + moxifloxacin → linezolid → meropenem → high-dose TMP-SMX; discharged on linezolid + TMP-SMX	Series-level data reported susceptibility to TMP-SMX; complete patient-specific panel not reported	Not reported	No recurrence at 6 months and 1 year
Miyoshi et al. 2026 [19]	64 / M	No relevant prior medical history; lung squamous-cell carcinoma diagnosed at autopsy	Lung, thyroid abscess	TMP-SMX → imipenem-cilastatin + levofloxacin	Susceptible: gentamicin, minocycline; resistant: penicillin, sulbactam-ampicillin, imipenem, cefazolin, cefotiam, cefotaxime, erythromycin, clindamycin, levofloxacin; TMP-SMX not tested	10-day hospitalization	Died on hospital day 10

Clinical presentation

Pulmonary nocardiosis may present with nodular, consolidative, or cavitary lesions and can resemble bacterial pneumonia, invasive fungal infection, mycobacterial disease, or septic pulmonary emboli [1,9,20-22]. Dissemination most commonly occurs hematogenously and may involve the CNS, skin, and other organs. In the present case, pulmonary infection was followed by recognition of a right lower extremity abscess and multiple intracranial lesions, resulting in the characteristic lung-brain-skin presentation.

Pulmonary involvement is the most common manifestation of nocardiosis [1,2,20,21,23]. CNS disease may present with focal neurologic deficits, seizures, altered mental status, or psychiatric symptoms such as hallucinations [1]. In some patients, however, intracranial involvement may initially be asymptomatic. Therefore, early brain imaging should be considered in immunocompromised patients with confirmed or strongly suspected nocardiosis, even when neurologic symptoms are absent.

Exposure typically occurs through inhalation of organisms from soil, although direct inoculation through cutaneous trauma may also occur [1,2,20,23-25]. A specific environmental exposure is not identified in many cases. Our patient reported past travel to Vietnam with possible soil exposure; however, the temporal relationship between this exposure and the subsequent infection cannot be established.

Risk factors

Nocardiosis has been reported in approximately 0.4-1.3% of renal transplant recipients [26,27]. Solid organ transplant recipients remain susceptible because glucocorticoids, calcineurin inhibitors, and other immunosuppressive agents impair T-cell function, phagocyte activation, and chemotaxis [27,28]. Impaired cell-mediated immunity may also contribute to susceptibility in patients with sickle cell disease [29]. Although the highest risk is often described during the early post-transplant period, cases may occur many years later. Our patient developed nocardiosis approximately 21 years after renal transplantation, which is consistent with reports describing onset from several weeks to more than two decades after transplantation [30].

Underlying pulmonary disease, particularly chronic obstructive pulmonary disease and bronchiectasis, may further increase susceptibility by impairing epithelial integrity and mucociliary clearance [20,21,23]. Nocardiosis can also occur in immunocompetent individuals and has been reported in up to one-third of patients in some series [1,23,31,32]. In patients without an apparent exposure or recognized immunocompromising condition, evaluation for an underlying immune defect should be considered.

Diagnosis

Disseminated nocardiosis can be difficult to distinguish clinically and radiographically from aspergillosis, mucormycosis, *Mycobacterium tuberculosis* infection, and *Staphylococcus aureus* infection with septic emboli [1,33]. A careful assessment of immune status, medication exposure, travel history, environmental exposure, and intravenous drug use may help narrow the differential diagnosis.

Definitive diagnosis relies on microbiologic isolation from respiratory specimens, tissue, abscess material, blood, or other involved sites. Modified acid-fast staining may demonstrate weakly acid-fast, branching filamentous organisms, while culture permits species-level identification and antimicrobial susceptibility testing [1,2,34,35]. Growth may be detected within several days but can require several weeks. Molecular identification is particularly valuable because susceptibility patterns vary among *Nocardia* species.

CNS involvement is associated with substantial morbidity and mortality. Accordingly, brain imaging should be obtained early in patients with disseminated disease or substantial immunosuppression, regardless of whether neurologic symptoms are present [1,35]. In our patient, brain MRI was obtained after the development of confusion and hallucinations and demonstrated multiple intracranial abscesses. Lumbar puncture was not performed, and the diagnosis of CNS involvement was based on the MRI findings in the setting of microbiologically confirmed disseminated nocardiosis.

Treatment

Treatment should be individualized according to disease severity, sites of involvement, antimicrobial susceptibility results, immune status, and treatment-related adverse effects. TMP-SMX remains a cornerstone of therapy, while alternative or additional agents include amikacin, linezolid, minocycline, imipenem, meropenem, ceftriaxone, amoxicillin-clavulanate, and selected fluoroquinolones [2,9,34-36]. Because susceptibility patterns are species-specific and may be unpredictable, treatment should be guided by formal susceptibility testing whenever possible [37].

Antimicrobial resistance among *Nocardia* species is heterogeneous and species-dependent. *N. otitidiscaviarum* has been reported to exhibit resistance to several beta-lactams, including amoxicillin-clavulanate and imipenem, and susceptibility to other antimicrobial classes may vary among isolates [3]. Although TMP-SMX is commonly used for nocardiosis, TMP-SMX-resistant *N. otitidiscaviarum* isolates have been reported, including fatal infections [37]. These variable resistance patterns reinforce the importance of species-level identification and isolate-specific susceptibility testing when selecting therapy.

Combination therapy is generally favored for pulmonary, CNS, disseminated, or life-threatening disease, whereas selected patients with localized cutaneous infection may be treated with monotherapy [35,38]. Prolonged treatment is usually required, particularly in immunocompromised patients and those with CNS involvement. Courses of at least 12 months are commonly used in these settings [1,9,35].

Our patient initially received multidrug therapy because of disseminated infection with intracranial involvement. TMP-SMX was discontinued after approximately one week because of worsening renal function and was reintroduced 15 days after the last dose after renal function improved. Linezolid was subsequently discontinued after hematologic abnormalities developed. Meropenem was also discontinued during the treatment course. The patient was discharged on TMP-SMX and minocycline and completed an initial six-week antimicrobial treatment course from the time of diagnosis. He was subsequently maintained on minocycline and moxifloxacin, with moxifloxacin later discontinued because of QT-interval prolongation. Minocycline was continued for slightly longer than one year, with clinical and radiologic resolution and no documented recurrence.

The patient was also treated for suspected pulmonary aspergillosis after BAL galactomannan testing was positive, although serum galactomannan and Fungitell assays were negative. No tissue culture or PCR testing for *Aspergillus* was obtained. Concurrent pulmonary aspergillosis and nocardiosis have been described in immunocompromised patients and may complicate diagnosis and treatment [2,39]. Because BAL galactomannan results must be interpreted in the context of the patient’s clinical presentation, imaging, host factors, and additional microbiologic findings, the diagnosis in this case is best described as suspected pulmonary aspergillosis based on the available documentation.

## Conclusions

Disseminated *N. otitidiscaviarum* infection is uncommon but should be considered in immunocompromised patients presenting with pulmonary disease accompanied by cutaneous or neurologic manifestations. This case highlights the importance of careful skin examination, early microbiologic diagnosis, species-level identification, and susceptibility-guided antimicrobial therapy. Because CNS involvement may initially be asymptomatic, early brain imaging should be considered in high-risk patients with confirmed or suspected nocardiosis. Prolonged treatment and follow-up imaging are essential to document clinical and radiologic resolution.
